# Achievement Goals and their Underlying Goal Motivation: Does it Matter Why Sport Participants Pursue their Goals?

**DOI:** 10.5334/pb.266

**Published:** 2016-07-13

**Authors:** Patrick Gaudreau, Arthur Braaten

**Affiliations:** 1 University of Ottawa, CA

**Keywords:** achievement goals, autonomous motivation, controlled motivation, performance, sport satisfaction, positive affect

## Abstract

This study examined whether the good or bad outcomes associated with mastery-approach (MAP) and performance-approach (PAP) goals depend on the extent to which they are motivated by autonomous or controlled motivation. A sample of 515 undergraduate students who participated in sport completed measures of achievement goals, motivation of achievement goals, perceived goal attainment, sport satisfaction, and both positive and negative affect. Results of moderated regression analyses revealed that the positive relations of both MAP and PAP goals with perceived goal attainment were stronger for athletes pursuing these goals with high level of autonomous goal motivation. Also, the positive relations between PAP goals and both sport satisfaction and positive affect were stronger at high levels of autonomous goal motivation and controlled goal motivation. The shape of all these significant interactions was consistent with tenets of Self-Determination Theory as controlled goal motivation was negatively associated with positive affect and sport satisfaction and positively associated with negative affect. Overall, these findings demonstrated the importance of considering goal motivation in order to better understand the conditions under which achievement goals are associated with better experiential and performance outcomes in the lives of sport participants.

Despite its ubiquitous nature in sport and life in general (Locke & Latham, 2013), goal setting is a multifaceted motivational strategy that can orient individuals toward different aims pursued for various reasons. Tenants of the Achievement Goal Theory (e.g., Elliot & McGregor, 2001) have provided ample evidence for the distinction between goals that are aimed at outperforming others (i.e., performance-approach; PAP) and those that are oriented at mastering tasks (i.e., mastery-approach; MAP). Despite an abundant literature in both the academic (e.g., Hulleman, Schrager, Bodmann, & Harackiewicz, 2010) and sport domains (e.g., Papaioannou, Zourbanos, Krommidas, & Ampatzoglou, 2012), the differentiated associations of PAP and MAP goals with performance and satisfaction/interest, respectively, remain to be fully elucidated. Willy Lens, with his work on the distinction between future goals that may underlie the pursuit of current goals (Lens, Simons, & Dewitte, 2002), inspired the last decade of research by his collaborators. His work laid the foundation for a novel line of work that illustrated the need to differentiate achievement goals and the reasons why individuals are pursuing them (Vansteenkiste, Lens, Elliot, Soenens, & Mouratidis, 2014). This research, based on basic premises of Self-Determination Theory (Deci & Ryan, 2002), proposes that the effects of achievement goals could be better explained after separating the aims (i.e., MAP and PAP) from their underlying reasons or goal motivation (i.e., autonomous and controlled goal motivation). In this study, we built upon the contributions and legacy of Willy Lens by examining whether autonomous and controlled goal motivation moderate the associations of MAP and PAP goals with the subjective experience (i.e., affective states, sport satisfaction) and perceived goal attainment of individuals participating in sport activities.

## Achievement Goals and their Differentiated Outcomes

In sport activities and life in general, individuals strive to perceive themselves as competent. Tenants of Achievement Goal Theory have, for long, proposed that distinct achievement goals can differentially orient, guide, and influence the behavior, cognition, and emotion of individuals in their quest to fulfill their need for competence (e.g., Elliot & Trash, 2001; Roberts, 2012). During the last decade, a growing number of researchers have defined and characterized achievement goals using a 2 × 2 framework that differentiates between the *definition* and the *valence* of achievement goals (e.g., Conroy, Elliot, & Hofer, 2003; Elliot & McGregor, 2001; Harackiewicz, Barron, Pintrich, Elliot, & Thrash, 2002). The definition refers to whether an achievement goal is considered task-based (i.e., developing a skill) or other-based (i.e., performing better than others). The valence refers to a distinction between an approach valence (focusing on attaining a desirable outcome) compared to an avoidance valence (focusing on avoiding a negative undesirable outcome). On the basis of a 2 × 2 matrix, this conceptual framework includes four subtypes of achievement goals: *mastery-approach* goals (MAP: aims to attain task mastery or improvement), *performance-approach* goals (PAP: aims to outperform others), *mastery-avoidance* goals (MAV: aims not to fall short of task mastery), and *performance-avoidance* goals (PAV: aims not to perform worse than others). Avoidance goals have been more frequently related to maladaptive outcomes in sport such as lower self-esteem, positive affect, satisfaction, performance, vitality, and self-confidence, as well as greater worry and anxiety (see review from Papaioannou et al., 2012). In this study, we were primarily interested in how athletes can achieve optimal levels of goal attainment and subjective experiences in their sport activities. Therefore, we decided to exclusively focus on two approach achievement goals: MAP and PAP.

Research has generally found that MAP and PAP goals are differentially associated with academic performance and satisfaction/interest (e.g., Harackiewicz, Barron, Pintrich, et al., 2002; Senko, Durik, & Harackiewicz, 2008). On the one hand, PAP goals tend to relate to higher levels of performance but not to interest and satisfaction. On the other hand, MAP goals have been significantly related to higher levels of interest and satisfaction but not to performance (e.g., Harackiewicz, Barron, Tauer, & Elliot, 2002; Pekrun, Elliot, & Maier, 2009; Verner-Filion & Gaudreau, 2010).

A meta-analysis of 19 studies performed in the sport domain by Lochbaum and Gottardy (2015) found that both MAP and PAP goals had a positive and moderate effect size with sport performance (*g* = 0.38 for both PAP and MAP). Similar results were reported in a second meta-analysis of 13 studies examining objective or non self-reported indicators of sport performance (Van Yperen, Blaga, & Postmes, 2014). In both meta-analyses, the relation between PAP goals and sport performance was heterogeneous across studies – a finding that suggests that further work is required to elucidate for whom and under which conditions PAP goals relate to better performance outcomes.

The relations between achievement goals and indicators of subjective experience has also receive substantial attention in sport research (e.g., Adie, Duda, & Ntoumanis, 2010; Hulleman, Durik, Schweigert, & Harackiewicz, 2008; Li, 2010; Morris & Kavussanu, 2009; Puente-Díaz, 2013). MAP goals have been significantly positively associated with increases in self-esteem and positive affect over time, greater hope, enjoyment, vitality, satisfaction, and pre-competitive self-confidence, as well as lower levels of worry, concentration disruption, and precompetitive somatic and cognitive anxiety. In contrast, these studies also have shown that PAP goals are unrelated to all of these outcomes, except for being positively related to hope and vitality. In addition, only PAP goals were associated with increases in negative affect over time. Overall, the findings in the sport psychology literature have provided robust evidence that MAP and PAP goals are distinctively related to affective states and satisfaction/interest of participants in sport settings.

## The Reasons or Underlying Goal Motivation of Achievement Goals

A novel stream of research has recently emerged and highlighted the importance of detaching the aims from the reasons underlying achievement goals (for a review, see Vansteenkiste, Lens, et al., 2014). This research has paralleled recent calls to conceptualize achievement goals in a narrower and more precise manner (Elliot & Murayama, 2008; Elliot & Trash, 2001; Vansteenkiste, Lens, et al., 2014) to focus on the aim or target of the goals rather than their underlying reasons or goal motivation. Allowing clearer separation of aims and reasons is desirable to investigate different “goal complexes” (Elliot & Trash, 2001). Numerous goal complexes are possible and each goal complex represents a specific combination of “aims” and “reasons”. In their qualitative study, Urdan and Mestas (2006) provided illustrious examples for the multiplicity of goal complexes with some students pursuing PAP goals (aim) to look smart to their parents (reason) while other students pursuing such aims for the enjoyment of competition (reason).

Studying goal complexes is a difficult task insofar as individuals can pursue the same achievement goal for a large number of reasons. Self-Determination Theory (Deci & Ryan, 2002) offers a promising theoretical framework to organize the reasons for pursuing goals into a smaller, yet parsimonious, number of conceptually and functionally distinct dimensions of motivation. Self-Determination Theory conceptualizes *autonomous motivation* as engaging in an activity for the pleasure and satisfaction that is inherent to it (intrinsic motivation), the importance that the person holds to it (identified motivation), or because it takes an integral part in the person’s life (integrated motivation). In contrast, *controlled motivation* refers to engaging in an activity in order to not feel guilty (introjected motivation) or in order to obtain something in return or avoid a negative consequence (external motivation).

In recent years, an increasing number of researchers have relied on Self-Determination Theory to investigate the motivation underlying personal goals (e.g., Koestner, 2008; Sheldon & Elliot, 1999) and achievement goals (e.g., Gaudreau, 2012; Michou, Matos, Gargurevich, Gumus, & Herrera, this issue; Vansteenkiste, Smeets, et al., 2010). Studies in the sport, education, and work domains have provided initial support for the proposition that goal motivations uniquely predict processes and outcomes, over and above the strength to which achievement goals are endorsed by participants (for a review, see Vansteenkiste, Lens, et al., 2014).

Three of these studies have examined the underlying reasons for achievement goals in the sport domain. Vansteenkiste, Mouratidis, and Lens (2010) examined autonomous and controlled reasons for pursuing PAP goals in male soccer players and reported that the reasons for pursuing PAP goals explained greater variance in well-being outcomes compared to endorsement of PAP goals. Autonomous goal motivation of PAP goals explained greater variance in soccer players’ positive affect and subjective vitality, whereas controlled goal motivation of PAP goals related to lower levels of positive affect and higher levels of negative affect. Vansteenkiste, Mouratidis, Van Riet, and Lens (2014) also performed a study in the sport domain using a within-person design to examine changes in volleyball players’ dominant achievement goal and their underlying goal motivation across six games. Although the dominant achievement goal did not predict a significant amount of unique variance in game enjoyment and performance satisfaction, the autonomous goal motivation of dominant MAP goals predicted greater game enjoyment and performance satisfaction. Similar findings were recently obtained in a study predicting the pre-competitive threat and challenge appraisals as well as the post-competitive self-talk, need satisfaction, flow, and performance of runners in a 20 kilometers race (Delrue et al., this issue).

## Supportive and Unsupportive Evidence for the “Aim” × “Reason” Effect

Despite mounting research interest, far less empirical support has been provided for the notion of goal complex. Goal complexes can be viewed as transactional constructs (Sameroff, 2009) wherein the “dynamic integration” of achievement goals and their accompanying goal motivation “essentially become intertwined … and work very closely together” (Elliot & Trash, 2001, see pp. 147–148). As eloquently summarized by Elliot and Trash (2001, p. 148), “in actual achievement settings the same goal may lead to somewhat different processes and outcomes, depending on its accompanying reasons”. The meaning or phenomenological experience of an achievement goal is likely to differ depending on the extent to which it is pursued for autonomous or controlled reasons. For example, a mastery goal coupled with an underlying autonomous motivation is likely to be experienced as a challenging, valued, and energizing endeavour. The same goal pursued out of controlled motivation is likely to be experienced as threatening, pressuring, and less desirable. On the basis of these arguments, it appears theoretically defendable to hypothesize that the same goal component (aim) should have a somewhat distinct “predictive profile” (Elliot & Trash, 2001, p. 148) depending on whether it is accompanied with an autonomous or a controlled goal motivation.

Examining the *moderating role of goal motivation* appears like a theoretically defendable approach to investigate goal complex. However, supportive evidence remains scant and inconsistent. A recent study with a large sample of Israeli middle school students (grade 7–8) provided indirect support by showing that having a sense of choice in a particular class – a proxy indicator of autonomy – significantly boosted the relation of MAP with both interest and behavioral engagement (Benita, Roth, & Deci, 2014). In a study with university students from France, Gillet, Lafrenière, Vallerand, Huart, and Fouquereau (2014) found that the relation between PAP goals and goal attainment was moderated by autonomous goal motivation. More precisely, PAP goals were most strongly related to greater goal attainment for students with higher compared to lower autonomous goal motivation. Still, this finding was not replicated in a second sample of first year students in the context of a police officer training camp.

Another stream of research has investigated multiple achievement goals rather than focusing on either MAP or PAP goals. Gillet, Lafreniѐre, Huyghebaert, Fouquereau (2015) performed three studies (two with undergraduate students from France and one with workers recruited on the internet) on autonomous and controlled reasons for pursuing each of the achievement goals. There was no perfectly consistent pattern of interactions across the three studies. Overall, autonomous motivation was a significant moderator between MAP goals and satisfaction, engagement, and positive affect in one sample; between PAP goals and engagement in two samples; and between PAP and satisfaction in one sample. On a similar note, the relation between MAP (but not PAP) and effort regulation (but not meta-cognitive and critical thinking) was moderated by autonomous goal motivation in a large sample of high school Greek students (Michou, Vansteenkiste, Mouratidis, & Lens, 2014).

Interestingly, the results of a study performed by Gaudreau (2012) with a sample of Canadian university students found some evidence for the moderating role of autonomous goal motivation for both MAP and PAP goals. In this study, MAP goals were significantly related to higher levels of academic satisfaction and performance for students who were pursuing these goals with higher self-concordant motivation (more autonomous reasons). Furthermore, PAP goals were significantly positively related to academic performance for students who pursued these goals with higher self-concordant motivation. These goals were negatively associated with academic satisfaction but only for students pursuing them with lower self-concordant motivation. Both MAP and PAP goals were also significantly related to higher levels of academic anxiety for students who were pursuing these goals with lower self-concordant motivation (more controlled reasons).

On the one hand, several studies have found limited supportive evidence for a statistically significant interaction between achievement goals and their underlying goal motivation (e.g., Delrue et al., this issue; Vansteenkiste, Smeets, et al., 2010). On the other hand, the supportive evidence reviewed above is nonetheless theoretically informative. In some situations, it appears like both MAP and PAP goals are more strongly related to positive achievement and psychological outcomes for individuals who are pursuing them with a higher level of autonomous goal motivation. The theoretically defensible *bolstering function of autonomous goal motivation* is worthy of further investigation.

## The Present Study

The objective of our study was to build on these findings by considering the underlying goal motivation of both MAP and PAP goals and their relations to selected sport outcomes such as perceived goal attainment, sport satisfaction, positive affect, and negative affect. These outcomes were specifically chosen because they represent “performance” and “quality of sport experience” that are hypothesized to be respectively associated with PAP and MAP goals.

Consistent with the premises of goal complexes (Elliot & Trash, 2001), we investigated whether the relations of MAP and PAP goals with sport outcomes are moderated by their underlying goal motivation (autonomous or controlled motivation). We expected MAP and PAP goals to show a distinct pattern of relationships with sport outcomes depending on the extent to which these goals were pursued with autonomous and controlled goal motivations. Despite the inconsistent findings in the literature, directional hypotheses can be formulated considering that autonomous goal motivation has been found to positively relate to a myriad of behavioral, cognitive, and emotion processes and outcomes in the Self-Determination Theory literature. High levels of autonomous goal motivation entail a sense of volition, agency, and empowerment that will likely lead to an increase in investment and sustained effort while alleviating the experience of emotional distress and ill-being (e.g., Koestner, 2008; Sheldon & Elliot, 1999). In contrast, high levels of controlled motivation indicate that goals are externally enforced and not deeply rooted or aligned with one’s true self. As such, autonomous goal motivation was expected to exhibit a bolstering function. More specifically, the positive associations of MAP and PAP with perceived goal attainment and positive sport experience (i.e., positive affect and satisfaction) were expected to be stronger for athletes with higher levels of autonomous goal motivation and either non-significant or significantly weaker for athletes with low levels of autonomous goal motivation and higher levels of controlled goal motivation.

## Method

### Participants

The study included 515 undergraduate students (68.5% females) who participated in sport competition, ranging from 17 to 48 years of age (*M* = 19.02, *SD* = 2.27) with 97% of the participants 24 years old or younger. Participants described themselves as Caucasian/White (75.3%), Asian (7.8%), African-Canadian/Black (4.7%), Arabic (3.3%), Aboriginal/Native (1.0%), Hispanic/Latino (0.4%), and other (1.7%). Students recruited were currently participating in sport competition at a recreational (46.8%), regional (30.8%), provincial (13.6%), national (7.4%), or international (0.8%) level from a wide range of different sports (e.g., hockey, basketball, track and field). In addition, athletes participated in their sport between 1 and 30 hours a week (*M* = 7.63, *SD* = 5.60). Participants provided informed consent and the study was approved by the institutional Research Ethics Board.

### Procedures and Measures

Participants were enrolled in a participation pool and received one point toward their introductory psychology class. They completed a 45–60 minute online survey alone at a time and place of their choice. In this study, *achievement goals and goal motivation* were both measured at the contextual level because athletes were asked to evaluate their overall experience in sport competition. In cross-sectional studies, measuring the independent variables at a broader level, while measuring the dependent variables at more specific level is useful to infer a top-down association between predictors and outcomes. It is also a good strategy to minimize the effect of shared method variance and overestimation of effect in cross-sectional designs (Podsakoff, MacKenzie, & Podsakoff, 2012). As such, participants referred to their latest sport competition in order to measure *goal attainment* as a situational state. Asking athletes to recall the affective states and sport satisfaction they experienced in their latest competition would have been prone to serious retrospective bias. Athletes were instead asked to evaluate the *affective states and sport satisfaction* they recently experienced about their sport. The specific instructions used to measure achievement goals, goal motivation, sport satisfaction, and sport affective states are presented hereafter.

### Measures

**Achievement Goals.** Achievement goals were assessed using two four-item subscales from the Orientation Sport Achievement Goals Scale (O-SAGS). These subscales were an adaptation from the School Achievement Goal Scale (SAGS; Verner-Filion & Gaudreau, 2010) for the sports domain. Participants were asked to indicate the extent to which each item represented the *“goals that you have when you are participating in a sport competition”.* The questionnaire stated, *“When I participate in sport competition, my goal is to …”* and contained four PAP (e.g., “*outperform other athletes*”, “*show that I am superior to other athletes*”) and four MAP (e.g., “*provide a quality effort*”, “*execute my movements correctly*”) achievement goal items. Participants rated the extent to which they agreed with each item on a scale from 1 (*not at all*) to 7 (*totally*). Gaudreau (2012) performed a confirmatory factor analysis in the academic domain and provided support for the hypothesized two-factor model (i.e., PAP and MAP). In the present study, Cronbach’s alpha was .92 for PAP goals and .88 for MAP goals.

**Underlying reasons of achievement goals.** The underlying reasons for pursuing achievement goals were tested in a similar method used in prior studies (Gaudreau, 2012; Vansteenkiste, Mouratidis, et al., 2010; Vansteenkiste, Smeets, et al., 2010). Participants were presented with four achievement goal statements. Two statements were used for MAP goals (i.e., “*In sport competition, athletes can try to execute their movement correctly and to provide a quality effort”*) and PAP goals (e.g., “*In sport competition, athletes can try to show that they are superior than other athletes and to outperform other athletes*”), respectively. For each of the four achievement goal statements, participants were asked to evaluate *“why do you pursue such a goal during a sport competition”* on a scale from 1 (*not at all for this reason*) to 7 (*totally for this reason*). The autonomous motivation items included an intrinsic reason (“*Because of the fun and enjoyment that this goal provides me*”) and an identified reason (“*Because it is important for my personal development*”). The controlled motivation items included an introjected reason (“*Because I put pressure on myself to have this goal*”) and an external reason (“*Somebody else is putting pressure on me*”). This method has been successful in evaluating the goal motivation of achievement goals in past studies (Gaudreau, 2012; Vansteenkiste, Mouratidis, et al., 2010; Vansteenkiste, Smeets, et al., 2010). In the present study, Cronbach’s alpha for autonomous motivation of PAP and MAP goals was .89 and .80, respectively. In addition, the alpha for controlled motivation of MAP and PAP goals was .87 and .90, respectively.

**Perceived goal attainment.** Goal attainment rather than objective sport performance was measured because it would be very difficult to create an index to compare and standardize the data across a wide range of different sports across heterogeneous levels of expertise. The Sport Achievement Goal Scale (A-SAGS; Amiot, Gaudreau, & Blanchard, 2004) is a 12-item questionnaire which contains three subscales measuring perceived achievement using absolute mastery-based (e.g., “*executed my movements correctly*”), normative performance-based (e.g., “*outperformed other athletes*”), and self-referenced intra-individual based (e.g., “*I did better than my usual performances*”) criteria of achievement. In this study, participants were asked to *“think about the last time you have participated in a competitive match, event, race, or tournament in your sport”.* Using the stem “*During the last competition, I…*”, participants were asked to evaluate the extent to which each item represented their performance during their last competition on a Likert scale from 1 (*not at all*) to 7 (*totally*). The A-SAGS results of a confirmatory factor analysis performed by Martinent, Nicolas, Gaudreau, and Campo (2013) confirmed that the three subscales of the A-SAGS can be used to form a general index of goal attainment. In this study, the three subscales were highly correlated (*r*s > .60). To remain consistent with all previous studies using the A-SAGS (e.g., Amiot et al., 2004; Gaudreau & Antl, 2008; Nicolas, Martinent, & Campo, 2014), the three subscales were averaged to create a global index. Cronbach’s alpha of this global score was .93.

**Sport satisfaction.** Sport satisfaction was measured with an eight-item subscale from the Multidimensional Student’s Life Satisfaction Scale (Huebner, Laughlin, Ash, & Gilman, 1998). Items were adapted to measure sport satisfaction (e.g., “*my sport is interesting”* rather than “*school is interesting”*). Athletes were asked “*In the last few weeks how do/did you feel about your sport?*” The participant rated the extent to which they agreed with each of the eight items on a Likert scale from 1 (*not at all agree*) to 7 (*totally agree*). Past research has shown acceptable internal consistency and a confirmatory factor analysis provided evidence for construct validity (Huebner et al., 1998). In addition, Gaudreau, Gunnell, Hoar, Thompson, and Lelièvre (2015) provided evidence that the adapted version of the MLSS in the sport domain had good reliability and good factorial validity. More precisely, the results of a confirmatory factor analysis, with a method factor accounting for negatively worded items, provided support for the good fit of the sport satisfaction subscale. Cronbach’s alpha for the scale in the present study was .78.

**Positive and negative affect.** Positive and negative affect was measured with the Positive and Negative Affect Schedule (PANAS; Watson, Clark, & Tellegen, 1988). The PANAS measures 10 positive affects (e.g., “*interested”*, “*excited*”) and 10 negative affects (e.g., “*distressed*”, “*upset*”). Athletes were asked “*In the last few weeks, how do/did you feel about your sport?*” and rated on a Likert scale from 1 (*very slightly or not at all*) to 5 (*extremely*). Watson et al., (1988) provided evidence for acceptable internal consistency and factorial validity. In addition, the PANAS has been widely and successfully used with samples of athletes (e.g., Gaudreau, Sanchez, & Blondin, 2006; Nicolas et al., 2014). Cronbach’s alpha was .89 for positive affect and .82 for negative affect.

### Plan of Analyses

Only three studies have simultaneously measured the goal motivation underlying both PAP and MAP (Gaudreau, 2012; Gillet et al., 2015; Michou et al., 2014) but different analytical strategies have already been used. On the one hand, Michou et al. (2014) have simultaneously included MAP and PAP goals in the same statistical analysis. However, they combined the goal motivation of MAP and PAP into a global goal motivation index – a justifiable decision in light of their strong correlation. On the other hand, the studies of Gaudreau (2012) and Gillet et al. (2015) have included each goal in a separate statistical analysis. At a first glance, this analytical decision might seem like a suboptimal approach considering the positive correlation between MAP and PAP goals. However, analyzing the two goals in the same regression analysis would force the inclusion of a total of 10 interrelated predictors (i.e., the two goals, four goal motivation variables, and four interaction terms). Considering that moderating effects are known to possess low statistical power (Shieh, 2009), keeping MAP and PAP in separate analyses was deemed preferable to favor parsimony, to avoid issues of multicollinearity, and minimize potential type II errors while facilitating the interpretation of the moderating effect. In the present study, the correlation between controlled goal motivation of PAP and MAP was large (*r* = .79, *p* < .001, see Table 1). Combining them into a global goal motivation index would have prevented us from studying “goal complex” and to test our research question. As such, analyzing PAP and MAP goals in separate analyses was a theoretically defensible compromise to ensure that each goal remained matched with its respective underlying goal motivation.

**Table 1:** Descriptive Statistics And Correlations.

	*M*	*SD*	1	2	3	4	5	6	7	8	9	10

1. PAP	4.82	1.57	–									
2. Autonomous motivation of PAP	4.46	1.60	.55***	–								
3. Controlled motivation of PAP	2.81	1.45	.26***	.23***	–							
4. MAP	5.95	0.93	.45***	.26***	.00	–						
5. Autonomous motivation of MAP	5.27	1.12	.21***	.45***	–.10*	.53***	–					
6. Controlled motivation of MAP	3.02	1.41	.22***	.13**	.79***	.03	–.09	–				
*Sport Outcomes*												
7. Perceived Goal Attainment	4.64	1.13	.34***	.30***	.08	.39***	.36***	.09*	–			
8. Satisfaction	5.90	0.74	.14**	.17***	–.18***	.43***	.42***	–.18***	.30***	–		
9. Positive Affect	3.87	0.70	.21***	.26***	–.06	.45***	.46***	–.06	.37***	.58***	–	
10. Negative Affect	1.68	0.55	.03	.01	.31**	–.22***	–.19***	.31***	–.04	–.22***	–.14**	–

*Note*. PAP = performance-approach goal; MAP = mastery-approach goal. * *p* < .05, ** *p* < .01, *** *p* < .001

Two separate set of multiple regression analyses were performed on (a) MAP goals and their goal motivation and (b) PAP goals and their goal motivation. In each of the two sets of analyses, we performed four hierarchical multiple regressions to predict one of the four dependent variables. Centered scores of goal endorsement (i.e., MAP or PAP) was entered at Step 1 whereas centered scores of the underlying autonomous and controlled goal motivations were entered at Step 2. At Step 3, we added the interaction terms between the goal endorsement and the goal motivation (e.g., MAP × autonomous motivation of MAP; MAP x controlled motivation of MAP) to test the moderating role of autonomous and controlled goal motivations. Significant interaction were examined using simple slope analyses at low (1*SD*) and high (+1*SD*) levels of goal motivation (Cohen, Cohen, West, & Aiken, 2003). Descriptive statistics and correlations are presented in Table 1,^1^.

## Results

### MAP goals (see Table 2)

**Table 2:** Moderated Regression Analyses Predicting Four Sport Outcomes on the Basis of Mastery-Approach Goals.

Model	Perceived Goal Attainment	Satisfaction	Positive Affect	Negative Affect

Step 1				
MAP	.39***	.43***	.45***	–.22***
*F* (1,513)	89.67***	114.43***	126.55***	26.68***
*R^2^*	.15	.18	.20	.05
Step 2				
MAP	.26**	.29***	.28***	–.20***
M-AUT	.23***	.25***	.31***	–.06
M-CON	.10**	–.16***	–.04	.31***
Change *F*	13.69***	28.30***	25.38***	31.34***
Change *R^2^*	.04	.08	.07	.10
Step 3				
MAP	.31***	.33***	.31***	–.17**
M-AUT	.21***	.25**	.30***	–.07
M-CON	.12**	–.16***	–.04	.35***
MAP X M-AUT	.10*	.07	.05	.08
MAP X M-CON	–.06	.01	.00	–.13**
Change *F*	4.11*	1.22	.80	7.07**
Change *R^2^*	.01	.00	.00	.02

*Note*. MAP = mastery-approach goal, M-AUT = autonomous motivation of MAP, M-CON = controlled motivation of MAP. * *p* < .05, ** *p* < .01, *** *p* < .001

**Figure 1:**
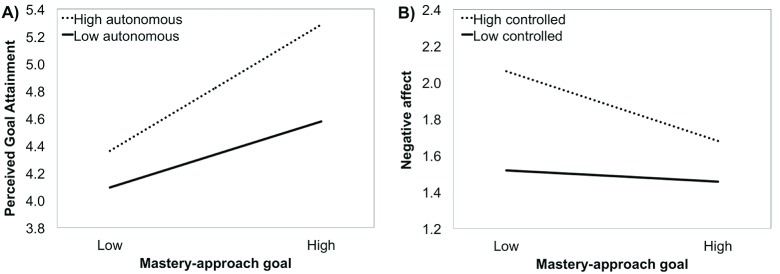
Moderating role of autonomous goal motivation in the relation between MAP goals and perceived goal attainment (Panel A) and moderating role of controlled goal motivation in the relation between MAP goals and negative affect (Panel B). Simple slopes estimated at –1*SD* and +1*SD* of the moderator.

At Step 1, MAP goals were significantly associated with perceived goal attainment, satisfaction, and positive affect while being negatively associated with negative affect. At Step 2, adding the goal motivation underlying MAP goals significantly increased the variance explained in all sport outcomes. Autonomous motivation for pursuing MAP goals significantly related to higher levels of perceived goal attainment, satisfaction, and positive affect. In contrast, controlled goal motivation of MAP goals was negatively associated with satisfaction and positively associated with negative affect and perceived goal attainment. At Step 3, the relation between MAP goals and perceived goal attainment was significantly moderated by autonomous goal motivation. As shown in Figure 1A, simple slope analyses revealed that MAP goals were positively and significantly associated with perceived goal attainment for those athletes who were pursuing these goals with high levels of autonomous motivation (*β* = .41, *p* < .001) and low levels of autonomous motivation (*β* = .21, *p* < .001). The relation between MAP goals and negative affect was also significantly moderated by controlled motivation (see Figure 1B). MAP goals were significantly negatively related to negative affect for athletes who were pursuing these goals out of high levels of controlled motivation (*β* = .35, *p* < .001), but not for those who pursued these goals with lower controlled motivation (*β* = .06, *p* > .05) 2.

### PAP goals (see Table 3)

**Table 3:** Moderated Regression Analyses Predicting Four Sport Outcomes on the Basis of Performance-Approach Goals.

Model	Perceived Goal Attainment	Satisfaction	Positive Affect	Negative Affect

Step 1				
PAP	.34***	.14**	.21***	.03
*F* (1,513)	65.49**	9.84**	23.40***	.41
*R^2^*	.11	.02	.04	.00
Step 2				
PAP	.25***	.11*	.13*	–.03
P-AUT	.17**	.17**	.22***	–.05
P-CON	–.02	–.24***	–.15**	.33***
Change *F*	5.91**	19.03***	13.36***	27.78***
Change *R^2^*	.02	.07	.05	.10
Step 3				
PAP	.26***	.13*	.15**	–.04
P-AUT	.20***	.21***	.27***	–.07
P-CON	–.03	–.27***	–.17***	.33***
PAP X P-AUT	.09*	.11*	.14**	–.07
PAP X P-CON	.04	.10*	.11*	–.03
Change *F*	3.22*	7.12**	10.93***	1.82
Change *R^2^*	.01	.03	.04	.01

*Note*. PAP = performance-approach goal, P-AUT = autonomous motivation of PAP, P-CON = controlled motivation of PAP. * *p* < .05, ** *p* < .01, *** *p* < .001

**Figure 2:**
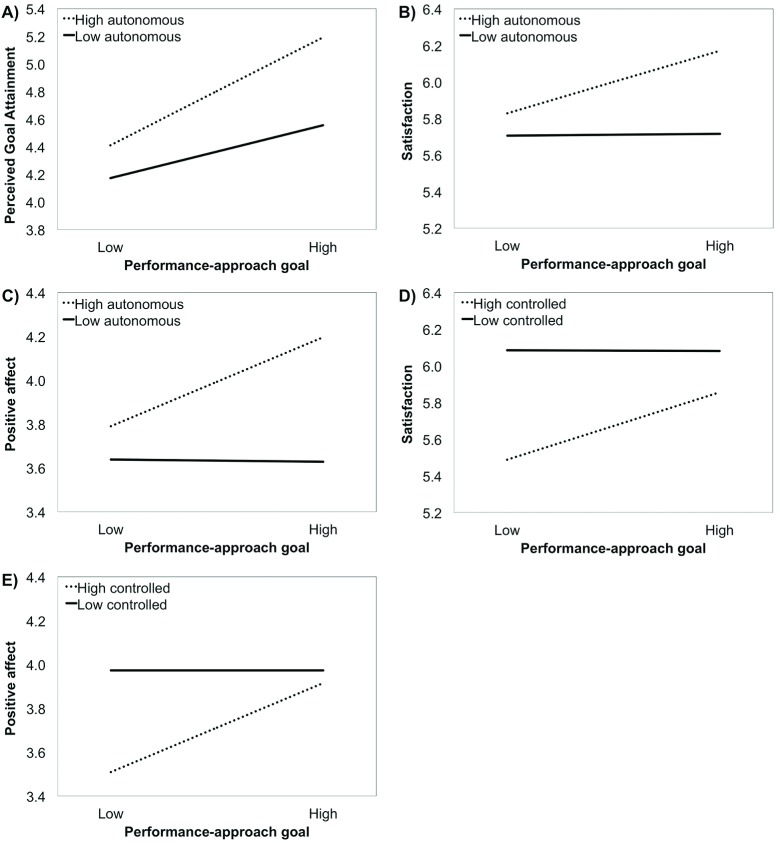
Moderating role of autonomous goal motivation in the relation between PAP goals and perceived goal attainment (Panel A), sport satisfaction (Panel B), and positive affect (Panel C) and moderating role of controlled goal motivation in the relation between PAP goals and sport satisfaction (Panel D) and positive affect (Panel E). Simple slopes estimated at –1*SD* and +1*SD* of the moderator.

At Step 1, PAP goals were significantly associated with higher perceived goal attainment, satisfaction, and positive affect. At Step 2, autonomous motivation for pursuing PAP goals related to higher levels of perceived goal attainment, satisfaction, and positive affect. In contrast, controlled goal motivation of PAP goals was negatively associated with satisfaction and positive affect while being positively associated with negative affect. At Step 3, autonomous motivation of PAP goals significantly moderated the relationship between PAP goals and perceived goal attainment, satisfaction, and positive affect. As shown in Figure 2A, the results revealed that PAP goals were significantly positively related to perceived goal attainment for athletes who were pursuing these goals out of high (*β* = .34, *p* < .001) or low (*β* = .17, *p* < .01) autonomous motivation. Furthermore, as shown in Figure 2B, PAP goals were significantly positively associated with satisfaction for athletes who were pursuing these goals out of high autonomous motivation (*β* = .23, *p* < .001) but not for those who pursued these goals out of low autonomous motivation (*β* = .01, *p* > .05). Finally, as shown in Figure 2C, PAP goals were significantly positively associated with positive affect for athletes who were pursuing these goals out of high autonomous motivation (*β* = .29, *p* < .001) but not for those who pursued these goals out of low autonomous motivation (*β* = .01, *p* > .05).

Controlled motivation of PAP goals also significantly moderated the relation between PAP goals and both satisfaction and positive affect. As shown in Figure 2D, PAP goals significantly positively related to satisfaction for athletes who were pursuing these goals out of high levels of controlled reasons (*β* = .25, *p* < .001), but not for those who were pursuing these goals out of low levels of controlled reasons (*β* = .00, *p* > .05). Similarly, as shown in Figure 2E, PAP goals significantly positively related to positive affect for athletes who were pursuing these goals out of high levels of controlled reasons (*β* = .29, *p* < .001) but not for those who were pursuing these goals out of low levels of controlled reasons (*β* = .00, *p* > .05)^3^.

## Discussion

Using a cross-sectional design, we examined both the *endorsement* (aims) and *underlying goal motivation* (reasons) of two types of achievement goals (MAP and PAP) in a relatively large sample of sport participants. Results of a series of multiple regression analyses – performed separately for the MAP and PAP goals – have shown that goal motivation can moderate the relation of achievement goal endorsement with perceived goal attainment, satisfaction, and affective states of sport participants. A total of seven “aim” × “reason” interactive effects reached statistical significance for goal attainment (two out of four), satisfaction (two out of four), positive affect (two out of four), and negative affect (one out of four). More specifically, autonomous goal motivation significantly bolstered the relation between MAP goals and *one of the four* outcomes (i.e., perceived goal attainment) and the relation between PAP goals and *three of the four* outcomes (i.e., perceived goal attainment, sport satisfaction, and positive affect). The bolstering function of autonomous goal motivation, along with the main effects of achievement goals and their underlying goal motivation, are discussed in light of their implications for both achievement goal and self-determination theories.

Differentiating “aims” and “reasons” – or goal endorsement and goal motivation – has been a *desideratum* of contemporary achievement goal theorists for the last 15 years (e.g., Elliot & Trash, 2001). In recent years, significant progress has been made by incorporating Self-Determination Theory to provide a theoretically driven framework to examine the goal motivation that underlies achievement goals (Vansteenkiste, Lens, et al., 2014). Important knowledge has been garnered because researchers have generally been able to show, replicate, and conclude that the autonomous goal motivation underlying the endorsement of MAP and PAP is associated with desirable outcomes (e.g., Benita et al., 2014; Gaudreau, 2012; Gillet et al., 2015; Gillet et al., 2014; Michou et al., 2014; Vansteenkiste, Mouratidis, et al., 2010; Vansteenkiste, Smeets, et al., 2010). Even mastery-avoidance goals (see Michou et al., this issue) and intrapersonal-approach goals (see Delrue et al., this issue) have the potential to be related to positive processes and outcomes for those who are autonomously motivated. Although achievement goal endorsement and their underlying goal motivation both seem to matter, we know far less about their combinatory, interactive or synergistic effects. *Goal complexes*, or the specific pairings of achievement goals and their underlying goal motivation, have been hypothesized to act as a transactional mechanism in which the underlying goal motivation presumably shapes and gives a distinct flavor to specific aims or goal endorsement (e.g., Elliot & Trash, 2001). Therefore, in this study, we focused our attention on the potentially moderating role of goal motivation in the relation of two types of achievement goals (MAP and PAP) with perceived goal attainment, satisfaction, and affective states of sport participants.

### Findings and Reflections on Mastery-Approach Goals

Consistent support has been found in the sport psychology literature for the positive association between MAP goals and satisfaction/interest (Papaioannou et al., 2012). In this study, MAP goals were positively associated with *sport satisfaction* and *positive affective states* and these relations were not significantly moderated by their underlying goal motivation. It thus seems that higher MAP goal endorsement and autonomous goal motivation are uniquely linked to positive feelings about sport participation. Higher levels of autonomous goal motivation for MAP goals, irrespective of the precise amount of goal endorsement (and vice versa), are operating independently to predict pleasurable feelings of engagement and satisfaction toward sport participation. These findings are similar to recent studies conducted with high school and university students in which MAP goal endorsement and autonomous goal motivation both significantly predicted self-regulated learning outcomes (Michou et al., 2014).

Results were somewhat different for the relation between MAP and *negative affect*, which was significantly moderated by controlled goal motivation (see Figure 1B). Unpleasant or distressful feelings about sport participation remained low for athletes pursuing their MAP goals with low levels of controlled goal motivation. In contrast, the relation between MAP goals and unpleasant affect was negative and significant for participants with higher levels of controlled goal motivation. For those sport participants, low level of MAP goal endorsement was associated with the highest levels of negative affect toward sport participation. It thus seems like lower endorsement of MAP goals coupled with higher controlled goal motivation creates a debilitative “goal complex” (Elliot & Trash, 2001) that fails to orient athletes toward task-based definition of competence while fuelling such goal endeavour with a sense of internal and external pressure likely to increase unpleasant affective states. The high negative affective states associated with such a goal complex is therefore consistent with postulates of both the Achievement Goal Theory and the Self-Determination Theory perspectives.

Results regarding the association between MAP goals and indicators of performance have been vividly debated among achievement goal theorists (e.g., Roberts, 2012). Proponents of a mastery goal perspective have hypothesized that MAP goals should systematically lead to desirable learning and achievement outcomes because such goals are likely to promote effortful task engagement and deep processing learning strategies. Results of two recent meta-analyses have revealed a positive and medium effect size of MAP goals on sport performance outcomes (Lochbaum & Gottardy, 2015;Van Yperen et al., 2014). In the current study, the relation between MAP goals and *perceived goal attainment* was significantly moderated by their underlying autonomous goal motivation (see Figure 2A). This finding, which is comparable to the results of Gaudreau (2012) with university students, showed MAP goals to be more strongly related to perceived goal attainment for sport participants who are pursuing them with high levels of autonomous goal motivation. Although the relation remained significant, it was significantly weaker for athletes with lower levels of autonomous goal motivation. Low levels of autonomous goal motivation attenuated the relation of MAP goals with perceived goal attainment in the current study whereas it buffered or reduced the relation to statistical non-significance in the study of Gaudreau (2012). Difference in sample size and measurement scheme (self-concordance index versus distinct scores of autonomous and controlled goal motivation) across the two studies warrants a prudent reinterpretation of this conclusion. Both the athletes in this study and the university students in the study of Gaudreau (2012) displayed comparably stronger effect of MAP goals when they were pursuing these goals with higher level of goal autonomy (athletes: *β* = 0.41; students: *β* = 0.49) compared to lower level of goal autonomy (athletes: *β* = 0.21; students: *β* = 0.10). As such, we are more confident in concluding that autonomous goal motivation could have a bolstering function likely to offer some non-negligible advantages to athletes insofar as their MAP goals are more strongly associated with perceived goal attainment.

### Findings and Reflections on Performance-Approach Goals

PAP goals showed an even more consistent pattern of association across three of the four outcomes measured in this study. Much discussion has surrounded PAP goals over the last decade because of their seemingly paradoxical positive association with performance outcomes combined with their negative or non-significant association with satisfaction/interest. In this study, we found that the associations of PAP goal endorsement with *perceived goal attainment, positive affective states, and sport satisfaction* were significantly moderated by autonomous goal motivation (see Figure 2, panel A to C). Across all three outcomes variables, the relation to PAP goals was positive and significantly stronger for the sport participants with higher levels of autonomous goal motivation. For athletes with lower levels of autonomous goal motivation, the relations of PAP goals to satisfaction and positive affect were non-significant whereas the relation with perceived goal attainment remained significant. These findings provided support for the theoretically expected bolstering function of autonomous goal motivation.

Our findings in regard to performance-related outcomes replicated the effects observed with university students across two previous studies (Gaudreau, 2012; Gillet et al., 2014). For example, both the athletes in this study and the Canadian university students in the study of Gaudreau (2012) displayed comparably stronger goal effects when they were pursuing their PAP goals with higher level of goal autonomy (athletes: *β* = 0.34; students: *β* = 0.52) compared to lower level of goal autonomy (athletes: *β* = 0.17; students: *β* = 0.15). These results indicate that goal attainment is higher when people have high PAP goals combined with high autonomous goal motivation. These findings are theoretically important because they contribute to an emerging stream of research trying to identify when and for whom PAP goals are associated with good rather than bad outcomes (Senko, Hulleman, & Harackiewicz, 2011). From an applied perspective, our results indicate that PAP goals are not inherently detrimental. In fact, PAP goals can be part of a “goal complex” that can be potentially beneficial as long as athletes are pursuing them for pleasure, importance, and in manners that are consistent with their values, interests, and priorities. It thus seems like the quest to obtain normative success can take a relatively positive flavor for individuals who are pursuing their PAP goals with autonomous goal motivation (Gaudreau, 2012; Gillet et al., 2014).

In Self-Determination Theory research, controlled goal motivation has typically been associated with negative outcomes (e.g., Miquelon & Vallerand, 2008; Vasalampi, Salmela-Aro, & Nurmi, 2009). In this study, the relations of PAP goals with both sport satisfaction and positive affect were significantly moderated by controlled goal motivation. The shape of these interactions, at a first glance, might seem counterintuitive because the positive association of PAP goals with sport satisfaction and positive affect only reached statistical significance at high level of controlled goal motivation. Hence, these two findings deserve further discussion to avoid misinterpretation.

A closer inspection of Figure 2 (see panel D and E) reveals three points worthy of discussion. First, the simple slopes indicate that athletes with low level of controlled motivation maintained high positive affect and sport satisfaction regardless of their PAP goal endorsement. Such a finding is consistent with Self-Determination Theory and with an abundant literature showing that individuals with low level of controlled motivation are generally doing better than individuals with high level of controlled motivation. Second, the specific shape of the significant interactions clearly indicates that pursuing PAP goals with high levels of controlled goal motivation is not associated with better affective states and sport satisfaction. Third, a combination of low PAP endorsement with external or self-imposed pressure was associated with the lowest sport satisfaction and pleasurable engagement in sport. One likely reason is in the sport domain, the normative quest to outperform competitors and to rank amongst the best is valued and reinforced insofar as it enables sport participants to be selected and to maintain themselves on the highest competitive levels. Low PAP endorsement combined with external or self-imposed pressure seems to create a “goal complex” in which the athletes are somewhat detached from the values and norms that are rewarded in competitive sport environments. Such a detachment could be the result of many social (i.e., controlling coaching environment) and contextual (i.e., performance setbacks) factors that would deserve further empirical examination to understand why different sport participants adopt the same achievement goals for different reasons.

### Additional Findings and Reflections

Despite our decision to focus on the moderating role of goal motivation, it appears reasonable to outline the fact that our findings have also provided strong support for the basic premises of Self-Determination Theory. As such, our findings are consistent with two studies published in this special issue (Delrue et al., this issue; Michou et al., this issue). How should we interpret the main effects of goal motivation in the context of a significant aims × reasons interaction? The main effect of goal motivation, in the context of a significant interaction, represents the association between the goal motivation and the outcome variable for prototypical sport participants with medium levels of achievement goal endorsement. In this context, autonomous goal motivation of achievement goals – either MAP or PAP – was positively associated with perceived goal attainment, sport satisfaction, and positive affect whereas controlled goal motivation was positively associated with negative affective states regarding one’s sport.

Over the last three decades, various streams of Self-Determination Theory research have shown that autonomy is a cardinal feature of psychological adjustment, growth, and flourishing (e.g., Vansteenkiste, Niemiec, & Soenens, 2010). In the context of setting and pursuing personal goals, researchers have repetitively demonstrated the benefits associated with goals that are pursued with a sense of volition, pleasure, ownership, and personal importance (e.g., Gaudreau, Carraro, & Miranda, 2012; Sheldon, 2014). Our findings suggest that achievement goals should probably be seen as a special case of personal goals in which the person aims at mastering task demands (MAP) and outperforming others (PAP) in the context of a specific achievement-related activity. Just like personal goals can be pursued for a variety of reasons, achievement goals – despite their close associations with the need for competence – can also be pursued with different levels of autonomous goal motivation. Achievement goals accompanied or stemming from higher amount of autonomous goal motivation might be experienced as more satisfying and pleasurable. They might also foster a deeper and more sustainable task engagement generally needed to attain learning, improvement, and satisfactory rankings in competitive achievement environments. Our results contribute to a growing theoretical and empirical attention that has been allocated to raise awareness to the fact that autonomy can provide the needed volitional strength to unpack the potential and minimize the potentially iatrogenic nature of achievement goals.

In the current study as well as in a recent study of Michou et al. (2014), a high correlation was observed between controlled motivation of MAP and PAP goals. We think that this correlation is attributable to at least two factors. First, there is a moderately high correlation between MAP and PAP. Hence, some athletes are potentially endorsing both MAP and PAP – which is something typically seen in the Achievement Goal Theory literature (Papaioannou et al., 2012). Motivation for one goal (e.g., MAP) might generalize or transfer to the motivation of the other goal (e.g., PAP) through a process of generalized goal endorsement. Second, controlled goal motivation emerges, in large part, from perceived social pressure (explicit pressure in external regulation; implicit or internalized pressure in introjected regulation). Social pressure is an exogenous factor that exists outside of the goal itself. As such, social pressure could potentially have a ubiquitous influence across all goals in a goal system regardless of the specific nature of the specific aim under consideration.

Transference of goal motivation across MAP and PAP is less likely to operate for autonomous goal motivation. Autonomous goal motivation is derived from the pleasure (i.e., intrinsic) and personal importance (i.e., identified) of the goal itself. Pleasure and importance are endogenous to the goal and they are more likely to influence one specific goal rather than all goals in a goal system. As autonomous motivation appears more likely to be idiosyncratic to a specific aim, future research should use distinct scores of autonomous and controlled motivation rather than combine them into relative autonomy or self-concordance index (e.g., Brunet, Gunnell, Gaudreau, & Sabiston, 2015; Koestner, 2008). Such an approach would help researchers in distinguishing different types of effects of autonomous and controlled goal motivation (e.g., unique, interactive, agreement, differentiation), particularly within the confines of polynomial regression analyses. In their recent polynomial regression analysis of personal goals, Brunet et al. (2015) showed that both agreement/similarity and positive ratio of autonomous and controlled goal motivation significantly predicted goal progress and self-reported grades of university students. Future work should start exploring different polynomial effects associated with the goal motivation underlying achievement goals.

Looking back at the extant literature to critically analyze and compare studies could potentially offer insights as to why the moderating role of goal motivation emerged in some studies and not in others. The bolstering function of autonomous goal motivation did emerge with some samples of university students, high school students, and sport participants. Hence, we doubt that *life domains* by themselves could explain the variability across studies. Conversely, *sample size* appears to be a critical study characteristic. A significant interaction emerged in studies relying on relatively large samples. The power to detect interactive effects is generally low insofar as these effects are estimated in fully saturated models that account for the main effect of the independent and moderating variables (e.g., Shieh, 2009). Furthermore, several of the interactions observed in this literature are characterized by an attenuation pattern (see Figures 1A and 2A) in which the relation between the achievement goal and the outcome remains significant at low levels of autonomous motivation. Such interactions are theoretically meaningful but harder to detect because they generally explain one or two percent of unique variance. Small samples could potentially lead to false negatives in which true interaction terms (i.e., strikingly non-parallel simple slopes at high and low levels of the moderator) could remain undetected. Conversely, casting a large net to recruit as many participants as possible could potentially create the condition to observe theoretically weak interaction terms (i.e., almost parallel simple slopes at high and low levels of the moderator). All being considered, conducting a priori power analysis would be commendable to optimize the likelihood of rejecting the null hypothesis while minimizing the risk of reporting statistically significant but theoretically weak interactive effects.

Looking forward to reflect upon potential areas of theoretical development appears just as important as looking back at the extant literature. Neither Achievement Goal Theory nor Self-Determination Theory was originally formulated to delineate the specific conditions under which goals and their underlying goal motivation should interact to predict desirable outcomes. Both theories have nevertheless delineated insightful principles to understand the social conditions under which individuals are more likely to develop and maintain their motivation. Feedback is required to help individuals to progress on and eventually attain their goals (e.g., Locke & Latham, 2013). Yet, not all type of feedback is sufficient to optimize the goal-related striving of individuals (e.g., Carpentier & Mageau, 2013; Mouratidis, Lens, & Vansteenkiste, 2010). As such, it appears reasonable to expect that the bolstering function of autonomous goal motivation is contingent on the type of support and feedback available in the social environment. Being in a class or a team that values and reinforces learning, effort, and mastery (rather than competition, ability, and talent) and receiving goal-directed support from autonomy supportive coaches, teachers, and parents might create the needed person × situation fit required for autonomous goal motivation to start playing its bolstering function. Without the support for mastery and autonomy, the interaction between the “aims” and the “reasons” might not reach statistical significance. Despite widespread scientific evidence showing their usefulness (e.g., Harwood, Keegan, Smith, & Raine, 2015; Vasquez, Patall, Fong, Corrigan, & Pine, in press), the support for mastery and autonomy are far from being the modal coaching and teaching style. Mastery climate and autonomy support are capable of promoting both the endorsement of achievement goals and their underlying autonomous goal motivation. What remains to be studied is whether mastery climate and autonomy support can transform and shape the dialectic relation between the “aims” and the “reasons” to offer the required nutrient for a goal complex to yield its most desirable outcomes.

### Limitations and Future Studies

The current study measured sport performance using a subjective self-reported rather than objective measure of sport achievement. Unfortunately, the use of an objective measure in this particular sample would have been impossible considering the wide range of sports and levels of expertise of the athletes recruited for this study. Every sport has a different set of motor skills that need to be mastered as well as a different system of ranking and performance assessment that also varies across gender and levels of competition. Although promising, the idea of measuring sport performance in a single sport is challenging insofar as a large sample would be required to offer a decently powered examination of the research question tested in the current study. A solution for future research would be to examine and focus on within-person variations rather than individual differences in sport performance (Dalal, Bhave, & Fiset, 2014). As far as we know Vansteenkiste, Mouratidis et al. (2014) is the only study that employed a within-person design examining achievement goals and their underlying reasons. A within-person model would look at within-person variations in goal endorsement, goal motivation, and performance across tasks, days, weeks, or performance episodes (e.g., competitions, exams), thus minimizing the need to recruit a homogeneous sample of athletes from a single sport, gender group, and/or level of expertise. For now, the current study offered a sufficiently powered test of the moderating role of goal motivation with a large sample of athletes from various sports, levels of expertise, and gender.

Our perceived goal attainment measure contained three interrelated subscales designed to capture different criteria used by individuals to evaluate their performance. Consistent with past studies, we created a global score to investigate perceived goal attainment (e.g., Amiot et al., 2004) because we did not formulate a specific hypothesis for each of the three subscales of goal attainment. In a study of high school students in physical education, Spray and Warburton (2011) showed that MAP and PAP goals displayed a similar pattern of correlations with mastery, self-referenced, and normative criteria of perceived competence. In this study, we focused on global attainment because conducting three separate multiple regressions was deemed suboptimal to take into account the strong correlations (*r*s > .60) and the proposed hierarchical organization of the subscales of goal attainment. Studying both global and specific criteria of goal attainment within the same statistical model would nonetheless be desirable to examine if the effect of a specific achievement goal can generalize across general and specific facets of goal attainment. As such, bi-factor analyses would be particularly helpful in future research to start investigating and potentially distinguish the nomological networks of global goal attainment and specific criteria of goal attainment (Myers, Martin, Ntoumanis, Celimli, & Bartholomew, 2014).

Two of the four PAP items used in this study are imperfect indicators of achievement goals because they contain some fragments of goal motivation (e.g., to show, to demonstrate). Researchers have recently advocated the use of newly created measures that neatly exclude goal motivation from the achievement goal items (e.g., Elliot & Murayama, 2008). Future research should try to replicate our findings using both the oldest and the newest measures of achievement goals. Participants could be randomly assigned in groups that would complete different questionnaires of achievement goals. An experimental design would enable a direct examination of whether results generalize across newest and traditional measures of achievement goals.

Finally, we did not simultaneously include the two types of achievement goals and their underlying goal motivation in the same multiple regression. Some researchers have showed that MAP and PAP can interact to influence key educational outcomes (e.g., Pastor, Barron, Miller, & Davis, 2007). Little is known about the reasons why some individuals are simultaneously pursuing both MAP and PAP goals whereas others are pursuing more specialized profiles of either MAP or PAP goals. Future work with large samples could explore the usefulness of sophisticated person-centered statistical models, such as cluster analyses and latent class models, to explore multidimensional “goal complexes” or interactions between all achievement goals and their respective underlying goal motivation.

## Conclusion

Overall, our results suggest the importance of considering the specific pairing between an achievement goal and its underlying goal motivation. Such pairings, called “goal complexes”, can be seen as a transactional mechanism in which the underlying goal motivation presumably shapes and gives a distinct flavor to the behaviors, cognitions, and emotions associated to an achievement goal. From an applied standpoint, these results suggest that setting MAP and PAP goals might not be sufficient to promote optimal goal attainment of competitive athletes, which potentially explains why not everyone benefit to the same extent from endorsing these goals. Setting MAP and PAP goals probably needs to be supplemented with interventions designed to ensure that these goals are consistent with one’s values, priorities, and interests. Whether such interventions can be delivered using simple and cost-effective writing activities (Yeager & Walton, 2011) or whether they would require the assistance of trained counselors or sport psychologists is an issue that warrants further investigation. Combining “aims” and “reasons” into distinct goal complexes amenable to randomized control trials is needed to provide stronger support for the non-causal effects observed in this correlational study and to generate evidence-based principles that could inform the daily work of applied sport psychologists.

## Footnotes

Participants were screened with a Mahalanobis distance calculated for each bivariate relation (cut-off *χ*^2^ = 13.816, *df* = 2, *p* < .001). Participants were removed (*n* = 15) before running the analyses when they were considered an outlier in the visual examination of the scatterplots and were also beyond the *χ*^2^. In addition, participants were removed if they answered less than two thirds of the items required to calculate a construct (*n* = 6).

Three complementary analyses were conducted on each of the three goal attainment subscales. Mastery attainment was significantly predicted (*p* < .05) by MAP (*β* = .46) and autonomous goal motivation (*β* = .22). Self-referenced attainment was significantly predicted (*p* < .05) by MAP (*β* = .16), autonomous goal motivation (*β* = .20), controlled goal motivation (*β* = .10), and the MAP × autonomous goal motivation (*β* = .12). Normative attainment was significantly predicted (*p* < .05) by MAP (*β* = .26), autonomous goal motivation (*β* = .15), controlled goal motivation (*β* = .13), and the MAP × controlled goal motivation (*β* = –.10).

Three complementary analyses were conducted on each of the three goal attainment subscales. Mastery attainment was significantly predicted (*p* < .05) by PAP (*β* = .19), autonomous goal motivation (*β* = .19), and the PAP × autonomous goal motivation (*β* = .11). Self-referenced attainment was significantly predicted (*p* < .05) by autonomous goal motivation (*β* = .20). Normative attainment was significantly predicted (*p* < .05) by PAP (*β* = .49) and autonomous goal motivation (*β* = .13).

## Author note

This research was supported by a Sport Canada Sport Participation Research Initiative grant from the Social Sciences and Humanities Research Council of Canada (862-2009-7) and a teaching release from the Faculty of Social Sciences awarded to Patrick Gaudreau.
